# Determining the Impact of COVID-19 on End-of-Life Experiences of Family Caregivers for People Living With Dementia

**DOI:** 10.1093/geroni/igab046.1246

**Published:** 2021-12-17

**Authors:** Sameer Mithani, Gwen McGhan, Deirdre McCaughey, Kristin Flemons

**Affiliations:** University of Calgary, Calgary, Alberta, Canada

## Abstract

COVID-19 has impacted all of our lives, but the population most at risk are older adults. Family caregivers (FCGs) for people living with dementia (PLWD) face challenges in providing care, which are compounded with the introduction of COVID-19 public health policies. The purpose of this study was to examine the experiences of FCGs where the PLWD died during the COVID-19 pandemic. FCGs were invited to participate in an online survey to examine their caregiving experiences during the COVID-19 pandemic, with the option of participating in a follow-up focus group. Sixteen FCGs whose family members with dementia died during the pandemic participated in the survey. A follow-up focus group was conducted to further examine how COVID-19 policies impacted their role as a caregiver in long-term care (LTC) and affected their ability to grieve. The results of the survey and focus group suggest that a lack of role clarity and inadequate communication channels between the FCG and LTC due to COVID-19 increased the strain FCGs faced during end-of-life care. At the end of life, public policies, such as reduced or no visitation, led to feelings of inadequacy and regret. Several participants also expressed appreciation for completing Advanced Care Planning documentation prior to COVID-19. Based on these results, policymakers can help ease the increased turmoil faced by FCGs during end-of-life care in future public health emergencies by involving FCGs of PLWD in the decision-making process. The completion of Advanced Care Planning documentation can also ease the burden FCGs may experience during end-of-life care.

